# Editorial: Advanced insights into environmental stress and tolerance mechanisms in forest trees

**DOI:** 10.3389/fpls.2023.1273663

**Published:** 2023-09-12

**Authors:** Rambod Abiri, Hazandy Abdul-Hamid, David WM Leung

**Affiliations:** ^1^ Department of Forestry Science and Biodiversity, Faculty of Forestry and Environment, Universiti Putra Malaysia (UPM), Serdang, Selangor, Malaysia; ^2^ Department of Plant Agriculture, University of Guelph, Guelph, ON, Canada; ^3^ Laboratory of Bioresource Management, Institute of Tropical Forestry and Forest Products, Universiti Putra Malaysia (UPM) Serdang, Selangor, Malaysia; ^4^ School of Biological Sciences, University of Canterbury, Christchurch, New Zealand

**Keywords:** forest trees, drought, salinity, cold, flooding, global warming, pest and diseases, gene and genomics

Forest ecosystems are beneficial green resources for maintaining ecological sustainability, economic stability, and social security, providing multiple advantages to both humanity and the environment. Nonetheless, forest environments face numerous threats and formidable challenges, including climate change, pathogen attacks, habitat degradation, and pollution. Therefore, understanding the defense mechanisms of trees in response to environmental stresses and their abilities for long-term survival remains crucial. It is critical to recognize how trees adapt to these environmental challenges, identify tree defense strategies, and understand how these strategies contribute to ensuring their long-term survival. Recent research, as shown in this Special Topic “Advanced Insights into Environmental Stress and Tolerance Mechanisms in Forest Trees”, revealed significant breakthroughs to help uncover specific responses and adaptation mechanisms of trees to environmental stressors. This topic outlines new opportunities for enhancing forest ecosystems as green resources.

He et al. assessed the influence of stand structure adjustment on biomass allocation and biomass accumulation as well as growth rate of *Pinus massoniana* seedlings. The investigation found that stand structure adjustment has a remarkable impact on the biomass pattern distribution in naturally generated *P. massoniana* seedlings. They found that seedlings in open stands were taller and developed more above ground biomass, while dense stands produced shorter seedlings with more subterranean biomass. Understanding such responses in *P. massoniana* helps to refine sustainable forest management practices by emphasizing the importance of environmental factors such as soil physiochemical properties, soil moisture, and light availability that influence development.

Liu et al. evaluated the regeneration of four dominant tree species, *Castanopsis fargesii*, *Lithocarpus glaber, Schima superba*, and *Hovenia acerba* seedlings, under the impact of understory filtering by the recalcitrant fern layer in a subtropical forest. The study demonstrated that filtering from the fern layer remarkably diminished the diversity and abundance of tree seedlings by limiting light availability and creating competition. They also found that some adaptive traits, such as optimal resource allocation approaches, deeper root systems, and shade tolerance enable tree seedlings to surmount the filtering effects. The results of this study highlight the importance of the regeneration stages, and that shade-tolerant species for predicting forest dynamics and implementing effective forest management and conservation approaches in subtropical forests with understory filtering.

Mayne et al. evaluated the dynamics of climate fluctuations on the resilience and growth rate of *Adansonia digitata* (tropical African baobab) at the range edge in climate-variable, southeast Africa over 90 years (1930–2020). Their team found that Baobab tree displays significant resilience and growth dynamics by adjusting their growth rate under unfavorable climates and enhancing steady growth during favorable climate conditions. This report also highlighted the legacy effects of past climate extremes, which increased African Baobab resilience to present-day fluctuations. Considering climate change, these findings underscore the relevance of hot droughts, climate variability, and landfalling tropical cyclones for Baobab management and conservation.

Vuosku et al. investigated the transcriptional responses of *Pinus sylvestris* (Scots pine) seedlings to different snow conditions and winter climate. The findings of this research demonstrate significant alteration in the expression of genes responsible for osmotic regulation, stress signaling, and protection against oxidative damage. Genes associated with hormone signaling pathways, growth, photosynthesis also displayed variation, suggesting resource allocation adjustments. Uncovering transcriptional response of Scots pine can aid in determining the best forest management strategies for shifting winter conditions.

Li et al. assessed precipitation patterns in relation to spring phenology in the Qinba Mountains, China. Evaluation of long-term data revealed that precipitation facilitates a more uniform timing of spring events. Winter and early spring precipitation reduces variability in budburst and leaf-out timing among species. They also observed a positive correlation between spring phenology and precipitation, with increased precipitation linked to earlier phenological periods. These findings have implications for ecosystem functioning and species interactions.

Zavadilová et al. studied the response of Norway spruce to long-term partial rainfall exclusion. Climate change affects precipitation patterns, which in turn, impacts tree growth and survival. The researchers excluded a portion of rainfall in a Norway spruce stand and monitored sap flow rates and growth parameters. They found that reduced rainfall significantly affected sap flow dynamics, indicating limited water availability for tree transpiration. Growth parameters, such as diameter increment and height growth, were negatively impacted by prolonged water deficit. The finding of this research emphasises the sensitivity of Norway spruce to changing precipitation patterns and indicates the importance of sufficient water availability for their vitality. Identifying their responses provides valuable insights for forest management strategies in the face of changing precipitation patterns associated with climate alteration.

Katsidi et al. evaluated the influence of genetics and epigenetic factors of *P. nigra* populations exposed to long-term air pollution. Using DNA sequencing and marker-based approaches, significant differences among plant populations with variable pollution exposure over the course of 70 years, suggesting possible adaptive responses. These results confirm the importance of genetic and epigenetic factors that influence tree population responses to air pollution, thus allowing pollution-tolerant specimens to be selected for reforestation efforts.

Budiadi et al. assessed the land cover changes, coastal development, biomass loss, and causes of a dieback event in a mangrove plantation using remote sensing data in Lampung, Sumatra. The dieback resulted in biomass loss, with declines in tree density and canopy cover. Understanding these changes and their causes is crucial for conservation and management strategies, including land use mitigation, mangrove restoration, and sustainable coastal development.

The diversity of trees in forests play a crucial role in maintaining ecological balance and providing benefits to the environment and humanity. However, vitality of the trees in the forests themselves is challenged by climate change, pollution, and habitat degradation. Recent studies in this Special Topic have shown progress toward understanding how forest trees can adapt to these stressors to enhancing their resilience. These findings contribute to forest conservation and management strategies in the face of environmental challenges.

## Author contributions

RA: Conceptualization, Supervision, Writing – original draft, Writing – review & editing. HH: Supervision, Writing – original draft, Writing – review & editing. DL: Conceptualization, Supervision, Visualization, Writing – original draft.

